# What's on the Plate? Unveiling Food Insecurity and Nutritional Risk Among Preschool‐Aged Children in Türkiye

**DOI:** 10.1002/fsn3.71676

**Published:** 2026-03-19

**Authors:** Gözde Dumlu Bilgin, Hasan Kaan Kavsara, İrem Kaya Cebioğlu, Aybüke Sarioğlu, Melis Keküllüoğlu Tan, Sema Aydin, Pınar Usta Ulutaş

**Affiliations:** ^1^ Department of Nutrition and Dietetics, Faculty of Health Sciences Yeditepe University İstanbul Turkey

**Keywords:** anthropometric indicators, food insecurity, nutritional risk, preschool children, socioeconomic factors

## Abstract

Despite growing attention to early childhood nutrition, limited research has investigated the connection between food insecurity and nutritional risk in preschool‐aged children. This cross‐sectional study aimed to evaluate the prevalence of nutritional risk using the Nutrition Screening for Toddlers and Preschoolers (NutriSTEP) tool and to investigate its association with household food insecurity and malnutrition. The study included 358 preschool children and their mothers, recruited from seven distinct Mother–Child Education Centers in Istanbul, Türkiye. Data collection included anthropometric measurements, the Household Food Security Survey‐Short Form, and the NutriSTEP questionnaire, along with maternal and household characteristics. Findings revealed that 31.6% of households experienced some degree of food insecurity. Children from food‐insecure households had significantly higher NutriSTEP scores (25.3 ± 7.0) compared to those from food‐secure households (23.4 ± 7.1, *p* = 0.018), indicating elevated nutritional risk. Children identified as high nutritional risk had significantly lower weight (17.8 ± 3.1 vs. 18.8 ± 2.9 kg, *p* = 0.002), height (105.4 ± 4.7 vs. 106.9 ± 4.3 cm, *p* = 0.003), mid‐upper arm circumference (MUAC), and waist circumference. Their z‐scores for weight‐for‐age, height‐for‐age, and weight‐for‐height were also significantly lower, and the prevalence of malnutrition was higher (4.2% vs. 0.4%, *p* = 0.016). Additionally, maternal education, parity, and perceived economic status were significantly associated with both food insecurity and nutritional risk. This study highlights the critical role of food insecurity in shaping early childhood nutritional outcomes, even without evident malnutrition. Combining NutriSTEP with food security tools supports early detection and intervention for at‐risk children.

## Graphical abstract

Nutritional Risk and Food Insecurity in Preschoolers.

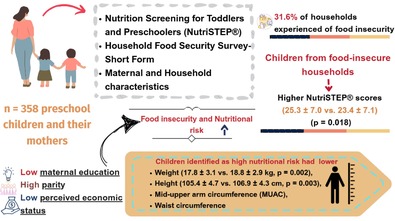

## 1 Introduction

Ensuring healthy nutrition in the early childhood period, including the prevention of malnutrition and adequate intake of essential macro‐ and micronutrients, is critical for optimal growth and development (Omand et al. 2021). The timely recognition of preschool children with poor eating patterns may contribute to reducing the likelihood of obesity and the onset of certain chronic diseases in adulthood (Ross and Kruger 2023).

Nutritional risk is identified as the existence of particular risk factors, such as abnormal eating behaviors, improper dietary intake, poor nutritional environment, and lack of food security, that may predispose to malnutrition (South et al. 2024). Nutrition screening can facilitate the identification of those at heightened nutritional risk by evaluating indicators of inadequate nutritional intake, lack of physical activity, and sedentary behaviors. These indicators may manifest prior to the recognition of weight fluctuations or the onset of nutrient deficiencies by healthcare professionals (Ross and Kruger 2023). The parent‐reported Nutrition Screening for Toddlers and Preschoolers (NutriSTEP) is a set of validated questionnaires designed to ascertain the nutritional risk of children between the ages of 18 months and 5 years. This scoring encompasses a range of nutritional status metrics, including the feeding environment, perceived weight status, dietary intake, eating behaviors, and food insecurity (Pulat Demir and Turgut 2022; Randall Simpson et al. 2008).

In recent years, food insecurity has become a salient component of nutritional risk and malnutrition in early childhood, thus establishing this issue as a prominent area of concern. According to the United Nations International Children's Emergency Fund (UNICEF) policy brief on the underlying factors of child malnutrition, household food insecurity, inadequate child care and unhealthy home environments, and lack of health services are the main drivers of child malnutrition (Black et al. 2008). The term “food security” is employed to denote the state in which all individuals within a given household possess sufficient, safe, and nutritious food that meets their dietary needs and food preferences (FAO 2014). Conversely, the term “household food insecurity” encompasses a range of experiences, including concern over food access and various degrees of food deprivation. It is imperative to note that all levels of household food insecurity, ranging from marginal to moderate to severe, persist as a global public health concern (Bayoumi et al. 2021; Shamah‐Levy et al. 2017; Wong et al. 2019). While early‐life exposure to food insecurity may be mitigated by parental protective measures, even marginal exposure to food insecurity has been associated with an elevated risk of suboptimal health outcomes, including nutritional deficiencies and the development of chronic health conditions in later life (El Bilbeisi et al. 2022; Wong et al. 2019).

Despite the emphasis on the significance of screening for nutritional risk in early childhood (Bayoumi et al. 2021; Omand et al. 2021), particularly the role of food insecurity (El Bilbeisi et al. 2022; Shamah‐Levy et al. 2017), few studies have simultaneously examined both the prevalence of nutritional risk and its relationship with household food insecurity, especially in urban low‐income settings. Household food insecurity may contribute to nutritional risk by limiting access to adequate and diverse foods, thereby affecting child growth and development. In this study, we hypothesized that nutritional risk as identified through the NutriSTEP framework would demonstrate a more robust correlation with anthropometric indicators in comparison to household food insecurity. This hypothesis was predicated on the conceptual distinction between the two constructs: NutriSTEP is a comprehensive assessment tool that evaluates various aspects of child nutrition and health, including dietary behaviors, feeding challenges, and nutrition‐related health concerns. It also considers the eating environment, which has been shown to have immediate effects on growth and nutritional status. Conversely, household food insecurity is indicative of broader economic food access at the family level. However, this may not result in measurable anthropometric differences in early childhood, potentially due to parental buffering strategies and protective feeding practices.

Therefore, the primary objective of this study was to elucidate the nutritional risk in preschool children using the NutriSTEP. The secondary objectives were to ascertain household food insecurity and assess its connection with nutrition risk and malnutrition in this population. By addressing these critical gaps in knowledge, this study provides valuable insights into the determinants of early childhood nutritional risk and food insecurity, with implications for the design and implementation of targeted nutrition interventions.

## 2 Materials and Methods

### 2.1 Participants and Data Collection

This cross‐sectional study was conducted as a continuation of the project previously initiated as the BESLEN Project (Dumlu Bilgin et al. 2024). The BESLEN project encompasses a series of studies on the nutritional status of various children and their mothers enrolled in seven distinct Mother–Child Education Centers within the Pendik district of Istanbul. These centers, operated by the municipality, offer a free service that hosts various children and their mothers during each academic year. The program incorporates a preschool education curriculum for the children, while the mothers participate in workshops focused on diverse hobbies.

The present multicenter study was conducted between October 2023 and June 2024. Participants were enrolled in two stages. First, following the acquisition of parental consent, researchers obtained anthropometric measurements from 358 preschool children registered in all participating centers. Subsequently, the researchers collected household food insecurity data and related sociodemographic questions, the NutriSTEP scale, and specific questions about maternal and child factors that may influence the child's nutrition through face‐to‐face data collection with the children's mothers. Consequently, a total of 716 subjects participated in the study, including 358 preschool children and their mothers.

This study was executed in accordance with the guidelines delineated in the Declaration of Helsinki, and all procedures were approved by the Marmara University Faculty of Health Science Non‐Interventional Research Ethics Committee (date and number: 26.10.2023/99).

### 2.2 Household Food Security Survey‐Short Form

The Household Food Security Survey Module (HFSSM), an 18‐item instrument, was developed in the United States in 1995 to assess household food insecurity. Subsequently, Blumberg et al. (1999) refined the instrument by selecting six items from the original 18, resulting in a more concise and efficient form. The Turkish validity and reliability of this questionnaire were examined by Öztürk Emiral et al. (2017). The scale is composed of six items that inquire about the adequacy of food intake in the previous 12 months, access to balanced meals, and the skipping of meals due to financial constraints despite being hungry. The participants were requested to respond to the inquiries by selecting one of the following options: For items 1, 2 and 4, they were asked to mark “Often true”, “Sometimes true”, “Never True”, “Don't know” or “Refused”; for items 3, 5 and 6, they were asked to mark one of the following options: “Yes”, “No” or “Don't Know”. The participant, who selected “Often True” for items 1, 2, and 4, and “Yes” for items 3, 5, and 6, received 1 point. All other responses received 0 points. The total score on the scale ranges from 0 to 6. Based on these scores, households were classified as food secure (score of 0), mildly food insecure (score of 1), moderately food insecure (scores of 2–4), or severely food insecure (scores of 5–6). To facilitate comparisons, these categories were dichotomized for statistical analyses into food secure versus food insecure (mild, moderate, or severe).

### 2.3 Nutrition Screening for Toddlers and Preschoolers (NutriSTEP)

The NutriSTEP was originally developed by Randall Simpson et al. (2008) to screen nutritional risks for children between the ages of 3 and 5. The validity and reliability of the Turkish version were examined by Pulat Demir and Turgut in 2022 (Pulat Demir and Turgut 2022). This tool was created to comprehensively assess children's nutritional intake, physical growth and development, physical activity and sedentary behavior, food safety, and the nutritional environment. The Turkish version of NutriSTEP is a 16‐item scale, with responses to inquiries 1, 2, 3, 4, 5, 11, and 15 scored on a scale of 0–4, and all other responses scored in reverse. The total score is calculated by aggregating the scores from each question. A high score indicates a high nutritional risk for children. In various studies, the scale's breaking points have been established as follows: ≤ 20 as low risk, 21–25 as medium risk, and ≥ 26 as high risk (Omand et al. 2021; Randall Simpson et al. 2008). However, in the Turkish version of the tool, the cut‐off values were observed to be similar to those of the Iranian population, with ≤ 27 designated as low risk, > 27 to ≤ 31 as medium risk, and > 31 as high risk (Mehdizadeh et al. 2020; Pulat Demir and Turgut 2022).

### 2.4 Anthropometric Measures

In the first phase of data collection, trained researchers measured the anthropometric parameters of children using standardized techniques and calibrated equipment. Body weight was measured using a portable digital scale with 100‐g sensitivity, and standing height was measured using a portable stadiometer accurate to 1 mm. Children were measured barefoot and wearing light clothing.

Mid‐upper arm circumference (MUAC) and waist circumference (WC) were measured to the nearest 0.1 cm using an inflexible measuring tape. The waist‐to‐height ratio (WHtR) was calculated by dividing waist circumference by height.

Children's nutritional status was assessed according to the World Health Organization (WHO) growth standards. WHO Anthro software was used for children under 5 years. Nutritional outcomes were classified based on *Z*‐scores: children with a height‐for‐age *Z*‐score (HAZ) ≤ −2 SD were considered stunting (chronic malnutrition), those with a weight‐for‐age Z‐score (WAZ) ≤ −2 SD were classified as underweight, and those with a weight‐for‐height *Z*‐score (WHZ) ≤ −2 SD were considered wasting (acute malnutrition) (World Health Organization 2017). Weight excess was assessed using WHZ with respect to > 1 SD cut point (Bejarano et al. 2019).

In the subsequent phase, data were collected in person from mothers regarding their children's birth characteristics, including birth weight, mode of delivery, and breastfeeding history, as well as sociodemographic factors, such as maternal age, education level, marital status, and parity. Birth weight was documented in grams, as recorded in health booklets.

### 2.5 Statistical Analysis

All statistical analyses were performed using IBM SPSS Statistics for Windows, Version 26.0 (IBM Corp., Armonk, NY, USA). Descriptive statistics were used to summarize the general characteristics of the participants. Continuous variables were presented as means ± standard deviations (SD) and ranges (minimum–maximum), while categorical variables were expressed as frequencies and percentages.

To examine differences in continuous variables between two independent groups (e.g., food secure vs. food insecure households; high vs. low nutritional risk according to NutriSTEP), independent samples *t*‐tests were used for variables that were normally distributed. The chi‐squared test (*χ* ^2^) was employed to assess associations between categorical variables. In instances where the anticipated cell counts were less than five, Fisher's exact test was employed instead of the chi‐squared test.

The classification of children into food‐secure and food‐insecure households was based on their responses to the HHFSSM‐SF. Nutritional risk was assessed using the NutriSTEP, with a total score > 27 indicating high nutritional risk (Pulat Demir and Turgut 2022).

Anthropometric indices, including WAZ, HAZ, and WHZ z‐scores, were calculated using WHO Anthro software. MUAC, WC, and WHtR were also compared across nutritional risk categories. A *p*‐value of < 0.05 was considered statistically significant.

To assess the association between food insecurity and NutriSTEP scores, multiple linear regression analyses were conducted. First, a crude model was constructed with food insecurity as the sole predictor to estimate the unadjusted association. Subsequently, adjusted models were developed to account for potential confounders: Model 1 has been adjusted for maternal education and household size. Model 2 has been thoroughly adjusted for maternal education, household size, birth order, and parity. Predictors were incorporated in a stepwise manner to illustrate the impact of potential confounders. For each model, the following metrics were reported: unstandardized regression coefficient (*B*), standard error (SE), 95% confidence interval (95% CI), standardized β and values, variance inflation factor (VIF).

## 3 Results

### 3.1 Household Characteristics of the Participants

The mean household size was 4.3 ± 1.1 members, ranging from 3 to 9 members. Most households (77.7%) had four or more members, while 22.3% had fewer than four. Regarding household income, 4.5% of families earned below the minimum wage, 39.4% earned at the minimum wage level, 38.0% earned between one and two times the minimum wage, and 18.1% earned more than twice the minimum wage. The self‐assessment of economic status indicated that 66.5% of families perceived their financial status as moderate, while 13.1% rated it as below average, 12.6% as above average, 6.1% as low, and 1.7% as high.

The household food security assessment showed that 68.4% of households were food secure, while 13.1% experienced mild food insecurity, 17.1% had moderate food insecurity, and 1.4% reported severe food insecurity according to the HHFSSM‐SF.

Regarding paternal employment, 89.7% of fathers were unemployed, while 6.7% were employed full‐time and 3.6% part‐time. The high proportion of unemployed fathers is consistent with the characteristics of the study population, which consisted of children and mothers attending municipality‐operated Mother–Child Education Centers providing free services. Although no official district‐level unemployment data for this specific population is available, this high rate is consistent with the socioeconomic characteristics of families. In terms of paternal education level, 22.6% of fathers had a university degree, 37.7% had completed high school, 23.5% had attended middle school, and 15.4% had finished primary school, with 0.8% being literate without formal education (Table 1).

**TABLE 1:** Household characteristics of the participants.

Household characteristics	
Members in the household (mean ± SD)	4.3 ± 1.1
(min.–max.)	(3–9)
< 4	80 (22.3)
≥ 4	278 (77.7)
Monthly income, *n* (%)	
Below minimum wage	16 (4.5)
Minimum wage	141 (39.4)
Between minimum wage and twice the minimum wage	136 (38.0)
More than twice the minimum wage	65 (18.1)
Self‐assessment of economic status, *n* (%)	
Low	22 (6.1)
Below average	47 (13.1)
Moderate	238 (66.5)
Above average	45 (12.6)
High	6 (1.7)
Household food security, *n* (%)	
Food secure (HHFSSM‐SF: 0)	245 (68.4)
Mild food insecurity (HHFSSM‐SF: 1)	47 (13.1)
Moderate food insecurity (HHFSSM‐SF: 2–4)	61 (17.1)
Severe food insecurity (HHFSSM‐SF: 5–6)	5 (1.4)
Employment status of father, *n* (%)	
Unemployed	321 (89.7)
Employed‐full time	24 (6.7)
Employed‐part time	13 (3.6)
Education level of father, *n* (%)	
University	81 (22.6)
High school	135 (37.7)
Middle school	84 (23.5)
Primary school	55 (15.4)
Literate	3 (0.8)

*Note:* Variables are shown as means ± standard deviations (SD) and minimum and maximum. Categorical variables are expressed as frequency (%).

Abbreviation: HHFSSM‐SF, Household Food Security Survey‐Short Form.

### 3.2 General Characteristics of the Participants

The study included 358 children, with 176 (49.2%) girls and 182 (50.8%) boys. The mean age of the children was 53.8 ± 3.2 months, ranging from 44 to 59 months. Regarding birth order, 39.9% of the children were first‐born, 33.2% were second‐born, 20.7% were third‐born, and 6.2% were fourth‐born or beyond. The mean birth weight was 3319.6 ± 610.5 g, ranging between 1250 and 5000 g. Most of the children (98.0%) had been breastfed, with mean breastfeeding duration of 19.1 ± 9.6 months, ranging from 1 to 55 months (Table 2).

**TABLE 2:** Characteristics of children by household food insecurity.

Characteristics	Food‐secure (*n* = 245)	Food‐insecure (*n* = 113)	Overall (*n* = 358)	*p*
*Children*				
Age, months	53.9 ± 3.0	53.7 ± 3.5	53.8 ± 3.2	0.597
	(47–59)	(44–59)	(44–59)	
Gender [boy, %]	122 (49.8)	60 (53.1)	182 (50.8)	0.561
Birth weight (g)	3305.7 ± 579.0	3349.7 ± 675.6	3319.6 ± 610.5	0.528
	(1300–5000)	(1250–4890)	(1250–5000)	
Birth order of the child [> 2, *n* (%)]	56 (22.9)	40 (35.4)	96 (26.8)	0.044*
Duration of breastmilk, months	19.4 ± 9.8	18.6 ± 9.1	19.1 ± 9.6	0.450
	(1.0–55.0)	(1.0–36.0)	(1.0–55.0)	
NutriSTEP total score	23.4 ± 7.1	25.3 ± 7.0	24.0 ± 7.1	0.018*
NutriSTEP assessment [high risk, *n* (%)]	73 (29.8)	45 (39.8)	118 (33.0)	0.061
Existing malnutrition, *n* (%)	5 (2.0)	1 (0.9)	6 (1.7)	0.669^a^
Weight excess, *n* (%)	70 (28.6)	33 (29.2)	103 (28.8)	0.902
Stunting, *n* (%)	5 (2.0)	1 (0.9)	6 (1.7)	0.669^a^
Underweight, *n* (%)	5 (2.0)	1 (0.9)	6 (1.7)	0.669^a^
Wasting, *n* (%)	2 (0.8)	0 (0.0)	2 (0.6)	NA
*Mother*				
Age, years	33.3 ± 5.2 (23.0–47.0)	33.4 ± 5.6 (22.0–48.0)	33.5 ± 5.4 (22.0–48.0)	0.439
Marital status [married, *n* (%)]	242 (98.8)	109 (96.5)	351 (98.0)	0.213^a^
Education [high school and more, *n* (%)]	155 (63.3)	45 (39.8)	200 (55.9)	< 0.001**
Mode of delivery [vaginal, *n* (%)]	98 (40.0)	52 (46.0)	150 (41.9)	0.283
Parity	2.1 ± 0.9	2.4 ± 1.0	2.2 ± 0.9	0.019*
	(1–6)	(1–5)	(1–6)	
Parity [> 2, *n* (%)]	68 (27.8)	43 (38.1)	111 (31.0)	0.050
*Household*				
Members in the household	4.2 ± 1.0	4.5 ± 1.1	4.3 ± 1.1	0.035*
Education level of father [high school and more, *n* (%)]	158 (64.5)	58 (51.3)	216 (60.3)	0.006**
Self‐assessment of economic status [low and below average, *n* (%)]	27 (11)	42 (37.2)	69 (19.3)	< 0.001**
Monthly income [between minimum wage and twice the minimum wage, *n* (%)]	105 (42.9)	31 (27.4)	136 (38.0)	< 0.001**

*Note:* Variables are shown as means ± standard deviations (SD) and minimum and maximum. Categorical variables are expressed as frequency (%). *p*‐values were computed by an independent *t*‐test for continuous variables and *χ* ^2^ for categorical variables.

Abbreviation: NutriSTEP, Nutrition Screening Tool for Every Pre‐schooler.

^a^ Fisher's exact test.

* *p* < 0.05.

** *p* < 0.01.

Among the mothers, the mean age was 33.5 ± 5.4 years, ranging from 22 to 48 years. The mean parity was 2.2 ± 0.9, with most mothers having two children (45.0%), followed by one child (24.0%), three children (22.9%), and four or more children (8.1%). Regarding mode of delivery, 58.1% of the children were born via cesarean section, while 41.9% were delivered vaginally. Educational status varied among mothers, with 19.6% having a university degree, 36.3% completing high school, 23.5% attending middle school, and 18.4% completing primary school. A small proportion was literate without formal education (0.8%) or illiterate (1.4%). Most mothers were married (98.0%), while 2.0% were single (Table 2).

### 3.3 Food Insecurity Conditions in the Last 12 Months

Food insecurity was assessed based on various conditions experienced by households in the 12 months preceding the study, as presented in Table S1.

*Insufficiency of nutrition intake*: About 6.4% of households reported that the food they purchased often did not last due to financial constraints, while 26.3% reported this as sometimes true. The majority (64.8%) stated that this was not an issue.

*Inability to afford balanced meals*: About 9.5% of households frequently could not afford balanced meals, while 33.0% experienced this problem sometimes. More than half (57.3%) reported no such issue.

*Reduction in meal size due to economic constraints*: About 18.4% of respondents indicated that they or other adults in the household had to reduce meal sizes or skip meals due to a lack of money for food, while 79.1% denied experiencing this problem. Among those who had reduced their meal size, 19.7% reported doing so every month, 68.2% in some months, and 10.6% for only 1 or 2 months.

*Eating less due to financial constraints*: 18.4% of respondents reported eating less than they felt they should due to financial limitations, while 79.6% did not experience this issue.

*Hunger without access to food*: 8.4% of respondents reported experiencing hunger without being able to eat due to financial difficulties, while 89.9% stated they did not encounter this issue.

### 3.4 Characteristics of Children by Household Food Insecurity

A key focus of this analysis was to compare NutriSTEP scores by food security status, given its relevance to nutritional risk. The comparison of children's characteristics based on household food security status revealed several significant differences, as presented in Table 2.

The mean age of children did not differ significantly between food‐secure (53.9 ± 3.0 months) and food‐insecure households (53.7 ± 3.5 months) (*p* = 0.597). Similarly, gender distribution (*p* = 0.561) and birth weight (*p* = 0.528) were comparable between the groups. However, children from food‐insecure households were more likely to be of higher birth order (> 2 children, 35.4%) compared to those in food‐secure households (22.9%, *p* = 0.044).

The duration of breastfeeding did not significantly differ between food‐secure (19.4 ± 9.8 months) and food‐insecure households (18.6 ± 9.1 months) (*p* = 0.450). Importantly, children from food‐insecure households had significantly higher NutriSTEP total scores (25.3 ± 7.0) than those from food‐secure households (23.4 ± 7.1, *p* = 0.018), indicating a greater nutritional risk associated with food insecurity. Although more children in food‐insecure households were classified as high‐risk by NutriSTEP (39.8% vs. 29.8%), the difference was not statistically significant (*p* = 0.061).

No significant differences were observed between food‐secure and insecure children in terms of malnutrition (*p* = 0.669), weight excess (*p* = 0.902), stunting (chronic malnutrition) (*p* = 0.669), underweight status (*p* = 0.669), or wasting (acute malnutrition).

The mean maternal age was similar between food‐secure (33.3 ± 5.2 years) and food‐insecure households (33.4 ± 5.6 years) (*p* = 0.439). Marital status also did not differ significantly (*p* = 0.213). However, a significantly higher proportion of mothers in food‐secure households had a higher level of education (63.3%), at least a high school diploma or higher, compared to mothers in food‐insecure households (39.8%, *p* < 0.001). Mothers from food‐insecure households had significantly higher parity (2.4 ± 1.0) than those in food‐secure households (2.1 ± 0.9, *p* = 0.019).

Households experiencing food insecurity had a significantly larger household size (4.5 ± 1.1 members) compared to food‐secure households (4.2 ± 1.0 members, *p* = 0.035). Education level of fathers was also significantly lower in food‐insecure households, with 51.3% having a high school education or higher, compared to 64.5% in food‐secure households (*p* = 0.006).

A significantly higher proportion of households experiencing food insecurity (37.2%) rated their economic status as low or below average, compared to 11.0% of food‐secure households (*p* < 0.001). This suggests that food‐insecure families were more likely to perceive their financial situation as inadequate compared to those in food‐secure households. Additionally, a significantly lower proportion of food‐insecure households (27.4%) reported earnings between minimum wage and twice the minimum wage, compared to 42.9% of food‐secure households (*p* < 0.001). This indicates that food‐insecure families had lower reported income levels, further supporting the association between economic hardship and food insecurity. Taking together, these results highlight that food insecurity is associated with a higher nutritional risk as reflected by significantly elevated NutriSTEP scores among children living in food‐insecure households.

### 3.5 Anthropometric Measurements of Children According to NutriSTEP Scores

The comparison of anthropometric measurements between children at high nutritional risk (NutriSTEP > 27) and those at low nutritional risk (NutriSTEP ≤ 27) revealed significant differences in several parameters as presented in Table 3. Children at high nutritional risk had a significantly lower mean weight (17.8 ± 3.1 kg) compared to those at low nutritional risk (18.8 ± 2.9 kg, *p* = 0.002). Similarly, their mean height was lower (105.4 ± 4.7 cm) than that of children at low nutritional risk (106.9 ± 4.3 cm, *p* = 0.003). Additionally, children at high nutritional risk had a significantly lower MUAC (17.0 ± 1.9 cm) compared to their low‐risk counterparts (17.4 ± 1.8 cm, *p* = 0.049). Waist circumference (WC) was also lower in the high‐risk group (53.1 ± 4.6 cm) compared to the low‐risk group (54.4 ± 4.0 cm, *p* = 0.004). However, no significant difference was observed in the waist‐to‐height ratio between the high‐risk (0.50 ± 0.04) and low‐risk groups (0.51 ± 0.03, *p* = 0.134).

**TABLE 3:** Anthropometric measurements of children according to NutriSTEP scores.

Measurements	High nutritional risk > 27* (*n* = 118)	Low nutritional risk ≤ 27 (*n* = 240)	Overall (*n* = 358)	*p*
Weight (kg)	17.8 ± 3.1	18.8 ± 2.9	18.5 ± 3.0	0.002**
Height (cm)	105.4 ± 4.7	106.9 ± 4.3	106.4 ± 4.5	0.003**
MUAC (cm)	17.0 ± 1.9	17.4 ± 1.8	17.2 ± 1.8	0.049*
WC (cm)	53.1 ± 4.6	54.4 ± 4.0	54.0 ± 4.3	0.004**
WHtR	0.50 ± 0.04	0.51 ± 0.03	0.51 ± 0.04	0.134
WAZ (*z*‐score)	−0.01 ± 1.21	0.51 ± 1.03	0.34 ± 1.12	< 0.001**
HAZ (*z*‐score)	−0.35 ± 1.15	0.09 ± 0.91	−0.05 ± 1.01	< 0.001**
WHZ (*z*‐score)	0.30 ± 1.22	0.69 ± 1.14	0.56 ± 1.18	0.003**
Existing malnutrition	5 (4.2)	1 (0.4)	6 (1.7)	0.016^a^, *

*Note:* Variables are shown as means ± standard deviations (SD). Categorical variables are expressed as frequency (%). *p*‐values were computed by an independent *t*‐test for continuous variables and *χ* ^2^ for categorical variables.

Abbreviations: HAZ, height/length for age *z*‐score; MUAC, Mid upper arm circumference; WAZ, weight for age *z*‐score; WC, waist circumference; WHtR, waist‐to‐height ratio; WHZ, weight for height/length *z*‐score.

^a^ Fisher's exact test.

* *p* < 0.05.

** *p* < 0.01.

Weight‐for‐age z‐score was significantly lower in the high‐risk group (−0.01 ± 1.21) compared to the low‐risk group (0.51 ± 1.03, *p* < 0.001). Height‐for‐age z‐score was also significantly lower in children with high nutritional risk (−0.35 ± 1.15) compared to those at low risk (0.09 ± 0.91, *p* < 0.001). Weight‐for‐height z‐score was significantly lower in the high‐risk group (0.30 ± 1.22) compared to the low‐risk group (0.69 ± 1.14, *p* = 0.003).

The prevalence of malnutrition was significantly higher among children at high nutritional risk (4.2%) compared to those at low nutritional risk (0.4%, *p* = 0.016). These findings indicate that children identified as high risk by NutriSTEP had lower anthropometric measurements and a higher prevalence of malnutrition compared to those classified as low risk.

### 3.6 Association Between Food Insecurity and NutriSTEP Scores

To examine the relationship between food insecurity and NutriSTEP score in further detail, multiple linear regression analyses were conducted. In the crude model, food insecurity exhibited a significant positive association with higher NutriSTEP scores (*B* = 1.917, 95% CI: 0.333–3.501, *p* = 0.018).

After controlling for confounding variables such as maternal education, household size, birth order, and parity, the association remained statistically significant (*B* = 1.646, 95% CI: 0.017–3.275, *p* = 0.048). A comparative analysis revealed that children from food‐insecure households displayed NutriSTEP scores ranging from 1.6 to 1.9 points higher than those from food‐secure households. The presence of multicollinearity was not detected, as evidenced by all VIF values being less than 1.1 (Table 4).

**TABLE 4:** Association between food insecurity and NutriSTEP scores.

Predictor	*B*	SE	95% CI	*β*	VIF	*p*
**Food insecurity**	—	—	—	—	—	—
Crude model	1.917	0.806	0.333–3.501	0.125	1.000	0.018*
Model 1 (Adjusted)	1.659	0.826	0.035–3.283	0.108	1.055	0.032*
Model 2 (Adjusted)	1.646	0.828	0.017–3.275	0.107	1.056	0.048*

*Note:* Multiple linear regression analyses were performed. The crude model included food insecurity only. Potential confounders were added stepwise: maternal education and household size (Model 1), plus birth order and parity (Model 2). Correlation is significant at *p < 0.05.

Abbreviations: *β* = standardized beta coefficient; *B* = unstandardized coefficient; CI, confidence interval; SE, standard error; VIF, variance inflation factor.

## 4 Discussion

Food insecurity has been a significant concern for global health in recent years, and it has the potential to lead to nutritional risks in early childhood. The aim of this study was to examine the prevalence of food insecurity and its related factors, and to investigate the relationship between food insecurity and nutritional risks and malnutrition among preschoolers. According to the World Food Program (WFP), more than 345 million individuals experienced high food insecurity in 2023. This alarming projection highlights the importance of Sustainable Development Goal (SDG) Target 2.1, which aims to end all forms of hunger by 2030 (Hussain et al. 2025). The findings of our study, conducted in Türkiye, reveal a remarkable prevalence of food insecurity among households (31.6%), with varying degrees of financial barriers to adequate nutrition associated with socio‐economic disadvantage. Although studies on the prevalence of food insecurity in Türkiye are limited, studies have reported a prevalence of 21.7%–30% in the elderly population (Simsek et al. 2013; Tari Selcuk et al. 2023b), 21.6% in women of childbearing age (Aytekin Sahin and Mengi Celik 2024), 24.4% in adults (Tari Selcuk et al. 2023a), and 33%–35.5% in university students (Celik et al. 2023; Ni̇yaz 2020).

In our study, we hypothesized that the prevalence of malnutrition would be higher among children in households experiencing food insecurity. A recent systematic review and meta‐analysis demonstrated that food insecurity is associated with an increased risk of stunting in children younger than 5 years (Moradi et al. 2019). Similarly, findings from another study revealed that children from food‐insecure households had lower average weight, height, and upper arm circumference measurements compared to their food‐secure peers (Qasrawi et al. 2024). However, contrary to these results, our findings did not show a relationship between food insecurity and the malnutrition status of preschool children, in line with some studies (Saaka and Osman 2013). The low prevalence of wasting and stunting in our sample may have limited our ability to detect significant differences in these outcomes. Moreover, this divergence can be attributed to several contributing factors. Malnutrition is influenced by variables beyond food access, including sanitation, infections, maternal factors, and caregiver practices (Vehapoglu et al. 2017). Food insecurity tools often measure perceived access rather than actual child intake or nutritional status. Furthermore, short‐term or seasonal food insecurity may not result in noticeable growth changes, particularly if households prioritize their children's nutritional needs. Although the HHFSSM‐SF assesses food insecurity over the past 12 months, seasonal variations can still influence household food access within that time frame, especially in regions where food prices, agricultural production, or employment patterns fluctuate across seasons (Vaitla et al. 2009). Therefore, seasonality was considered as a potential contributor to short‐term fluctuations in food availability, even if captured within a 12‐month recall period. Coping strategies and external support can also mitigate the effects (Saaka and Osman 2013).

The observed discrepancy, wherein anthropometric differences were not identified across food security status yet were significant across NutriSTEP categories, may be explained by the conceptual pathways through which these factors influence child growth. NutriSTEP is a comprehensive assessment tool that evaluates proximal, child‐specific determinants of nutrition. These determinants include dietary quality, feeding practices, eating environment, and nutrition‐related health behaviors. Collectively, these factors influence growth and body composition, with the potential to exert immediate and measurable effects. Conversely, household food insecurity is a distal socioeconomic determinant that reflects broader economic access to food at the family level. It has been demonstrated that young children may be shielded from the immediate consequences of food insecurity as a result of protective parenting strategies, the prioritization of children's food allocation, and the utilization of support systems, such as relatives, community programs, and government social aid. Consequently, the manifestation of household‐level food insecurity may not be immediate, as evidenced by anthropometric deficits in early childhood. In contrast, the assessment of nutrition risk utilizing the NutriSTEP method exhibits heightened sensitivity to early alterations in dietary behaviors and feeding dynamics.

Although food insecurity is not directly reflected in the results regarding malnutrition, it is a noteworthy finding in light of its association with NutriSTEP scores, an important nutritional risk assessment tool in our study. Prior studies employing the NutriSTEP tool have estimated that the prevalence of nutrition risk ranging from moderate to high is between 19% and 45% in early childhood (Randall Simpson et al. 2008). According to the NutriSTEP assessment, approximately 33% of the children in our study were classified as being at high nutritional risk. Reported prevalence rates of nutritional risk assessed using the NutriSTEP tool vary internationally, ranging from 19% in New Zealand (Ross and Kruger 2023) to 12% moderate and 28.6% high risk in Iran (Mehdizadeh et al. 2020), 16% in Canada (Omand et al. 2021), and as high as 21.1% moderate and 45.3% high nutritional risk in Türkiye (Pulat Demir and Turgut 2022). Such differences may stem from variations in socioeconomic status, access to healthcare, and cultural feeding practices among different regions. In addition, it should be noted that the thresholds used to define nutrition risk in this study differ from those applied in other countries, meaning that some observed differences may reflect methodological variations rather than actual disparities in nutritional status.

Notably, findings from our study indicate that children living in food‐insecure households had significantly higher NutriSTEP scores, reflecting an increased susceptibility to nutritional risk. Following the adjustment for potential confounders, including maternal education, household size, birth order, and parity, children from food‐insecure households still exhibited higher NutriSTEP scores. This finding indicates that food insecurity independently contributes to increased nutritional risk. Furthermore, the NutriSTEP questionnaire includes a specific item addressing food insecurity: “I have difficulty buying the food I want to feed my child because food is expensive.” This item has been validated as a reliable proxy indicator for identifying household food insecurity within the context of nutritional risk screening (Bayoumi et al. 2021). Also, children from food‐insecure households have been shown to be more prone to have poor diet quality (Chiu‐Wen et al. 2021), consume fewer fruits and vegetables (de Araújo et al. 2018; Hanson and Connor 2014; Landry et al. 2019), consume more processed foods (Pilgrim et al. 2012; Ribas et al. 2025), and have growth or weight problems (Kral et al. 2017). All of these factors can lead to higher risk scores on the NutriSTEP screening tool. Supportively, in our study, 9.5% of households frequently and 33.0% occasionally could not afford balanced meals. Alarmingly, 18.4% of adults reported that they were reducing or skipping meals, and a similar proportion stated they ate less than they felt they should due to limited financial resources. A recent review study highlights the importance of this issue by stating that food insecurity is strongly associated with poor nutritional outcomes, including stunting, undernutrition, and micronutrient deficiencies (e.g., vitamin A, iron, zinc), and has a detrimental effect on child development, resulting in growth deficits, cognitive impairments, and socioemotional difficulties (de Paiva et al. 2025). Therefore, our findings require cautious interpretation, with awareness of the long‐term implications of food insecurity on child development.

Educational status also emerged as a critical determinant of food insecurity, revealing that lower levels of education can lead to an increased risk. Our findings are consistent with international findings. For example, Basiry et al. (2024) reported that in Afghanistan, food insecurity among school‐aged girls was significantly more prevalent in households where parents, especially mothers, had lower educational attainment (Basiry et al. 2024). Similarly, Encalada‐Torres et al. (2022) demonstrated a direct link between low income and malnutrition among older adults in Ecuador (Encalada‐Torres et al. 2022). Supporting this, a meta‐analysis by Besora‐Moreno et al. (2020) confirmed that individuals with lower income levels had a significantly higher risk of food insecurity (Besora‐Moreno et al. 2020). In the United States, Walker et al. (2021) also emphasized a strong income‐based gradient in food insecurity (Walker et al. 2021).

Our study revealed that mothers from food‐insecure households had higher parity compared to those from food‐secure households, indicating a potential association between increased birth events and heightened vulnerability to food insecurity. Because we also found that households with more members are more food‐insecure than smaller households. These findings align with the study conducted by Ware et al. (2021) in an urban African township, where higher parity was linked to increased social vulnerability and greater exposure to food insecurity among young women. The authors emphasized that as parity increased, so did the indicators of socioeconomic hardship, further exacerbating food insecurity risk (Ware et al. 2021). Conversely, findings from Hasan et al. (2021) contradicted this association, reporting no statistically significant differences in parity distribution between food‐secure and insecure groups, suggesting that parity alone may not entirely predict food insecurity risk (Hasan et al. 2021). However, reinforcing our results, Ujah et al. (2023) found that in Nigeria, having five or more birth events significantly increased the odds of food insecurity among pregnant women, especially when accompanied by a larger household size (Ujah et al. 2023). The researchers highlighted that increased parity contributes to higher household food demands and economic burden, thereby reducing the ability to secure adequate nutrition. These inconsistencies across studies may reflect regional and contextual differences in access to family planning, socioeconomic resources, and healthcare services.

Our study has significant strengths and limitations that should be considered when interpreting the findings. A key strength lies in its focus on a critical public health issue by examining the relationship between household food insecurity and nutritional risk in preschool‐aged children, a population often underrepresented in current literature. The use of two validated instruments, the HHFSSM‐SF and NutriSTEP, enabled a comprehensive and reliable evaluation of both food security status and nutritional risk. Furthermore, the inclusion of broad sociodemographic and anthropometric data allowed for a multifaceted analysis of potential risk factors. The relatively large and diverse sample size also strengthens the general relevance and internal validity of the results within the studied context. However, several limitations must be acknowledged. The cross‐sectional design restricts the ability to infer causality between food insecurity and associated factors such as parity or maternal education. The reliance on self‐reported data may have introduced recall or social desirability bias. Additionally, the HHFSSM‐SF effectively captures household perceptions of food access, but it does not directly measure dietary intake or nutritional status, which may partly explain the non‐significant associations with malnutrition indicators.

## 5 Conclusion

In conclusion, while food insecurity did not correlate with direct anthropometric deficits in this preschool sample, it was strongly associated with increased nutritional risk as captured by the NutriSTEP screening tool. This underscores the importance of employing comprehensive assessment tools that capture both tangible and perceived nutrition‐related challenges. Public health interventions should prioritize families experiencing food insecurity by integrating targeted nutrition education and economic support programs. Future longitudinal studies should be conducted to determine how persistent food insecurity influences growth trajectories and developmental outcomes in early childhood.

## Author Contributions

**Gözde Dumlu Bilgin:** conceptualization (equal), formal analysis (equal), investigation (equal), methodology (equal), project administration (lead), supervision (lead), visualization (equal), writing – original draft (lead). **Hasan Kaan Kavsara:** conceptualization (equal), formal analysis (equal), investigation (equal), methodology (equal), visualization (equal), writing – original draft (lead). **İrem Kaya Cebioğlu:** conceptualization (equal), investigation (equal), methodology (equal), project administration (lead), supervision (lead), writing – original draft (supporting). **Aybüke Sarioğlu:** data curation (equal), investigation (equal). **Melis Keküllüoğlu Tan:** data curation (equal), investigation (equal). **Sema Aydin:** data curation (equal), investigation (equal). **Pınar Usta Ulutaş:** data curation (equal), investigation (equal).

## Funding

The authors have nothing to report.

## Conflicts of Interest

The authors declare no conflicts of interest.

## Supporting information


**Table S1:** Percentage of households experiencing certain food insecurity conditions in the last 12 months prior to the study.

## Data Availability

The data that support the findings of this study are available on request from the corresponding author. The data are not publicly available due to privacy or ethical restrictions.
