# Supplementary material for: What's on the Plate? Unveiling Food Insecurity and Nutritional Risk Among Preschool‐Aged Children in Türkiye
Source: Food Sci Nutr. 2026 Mar 19;14(3):e71676. doi: 10.1002/fsn3.71676 (PMC13093616; doi:10.1002/fsn3.71676)
Supplement: Supplementary file 1 — Table S1: Percentage of households experiencing certain food insecurity conditions in the last 12 months prior to the study. [file FSN3-14-e71676-s001.docx]

**Supplementary Table S1**: Percentage of households experiencing certain food insecurity conditions in the last 12 months prior to the study

| **Factors/Questions** | **Often true**  **n (%)** | **Sometimes true**  **n (%)** | **Not true**  **n (%)** | **Don’t know**  **n (%)** |
| --- | --- | --- | --- | --- |
| ***Insufficiency of nutritional intake***   1. The food that we bought just didn’t last, and we didn’t have the money to get more | 23  (6.4) | 94  (26.3) | 232  (64.8) | 9  (2.5) |
| ***Not assessing a balanced meal***   1. We couldn’t afford to eat balanced meals. | 34  (9.5) | 118  (33.0) | 205  (57.3) | 1  (0.2) |
| ***Reducing the size of the meal because of economic inadequacy***   1. Did you or other adults in your household ever cut the size of your meals or skip meals because there wasn’t enough money for food? | **Yes**  **n (%)** | **No**  **n (%)** | **I don’t know**  **n (%)** | |
|  | 66 (18.4) | 283  (79.1) | 9  (2.5) | |
| **If yes;**  The frequency of reducing the size of meals because of economic inadequacy. | **Every month**  **n (%)** | **Some months**  **n (%)** | **1 or 2 months**  **n (%)** | **I don’t know**  **n (%)** |
|  | 13 (19.7) | 45  (68.2) | 7  (10.6) | 1  (1.5) |
| ***Eating less because of economic inadequacy***   1. Did you ever eat less than you felt you should because there wasn’t enough money for food? | **Yes**  **n (%)** | **No**  **n (%)** | **I don’t know**  **n (%)** | |
|  | 66 (18.4) | 285  (79.6) | 7  (2.0) | |
| ***Could not eat even he/she is hungry because of economic inadequacy***   1. Were you ever hungry but didn’t eat because there wasn’t enough money for food? | 30  (8.4) | 322  (89.9) | 6  (1.7) | |

Categorical variables are expressed as frequency (%).
